# Complex Proximal Humeral Fracture Fixation with PHILOS Plate using Minimal Invasive Percutaneous Plate Osteosynthesis (MIPPO) Technique: A Series of 30 Patients

**DOI:** 10.5704/MOJ.1807.004

**Published:** 2018-07

**Authors:** VL Narayanan, N Balasubramanian

**Affiliations:** Department of Orthopaedics, Saveetha Medical College and University, Chennai, India

**Keywords:** proximal humerus fracture, locking compression plate, MIPPO technique, DASH score

## Abstract

**Introduction:** Proximal humerus fracture fixation using plate osteosynthesis depends on the quality of the bone, design of the fixation devices and intra-operative soft tissue dissection. This study evaluates the functional outcome of minimally invasive percutaneous plate osteosynthesis using locking compression plate in proximal humerus fracture treatment.

**Materials and Methods:** The study was conducted on 30 patients with complex proximal humerus fractures treated by minimally invasive percutaneous plate osteosynthesis using locking compression plate (PHILOS). There were 21 males and 9 females. The average age of our study group was 58.8 years. All the patients were evaluated at six weeks, three months, four months, six months and 12 months following surgery.

**Results:** All patients had fracture union at an average of 13.2 weeks. The mean DASH score at the follow-up was 8.69 (2.5 to 17.16), the average range of flexion was 143.83 degrees (100 to 170 degrees) and abduction was 121.49 degrees (90 to 160 degrees). We had superficial infection in three patients which resolved with a short course of antibiotics. There was excellent outcome in 26 patients, good and fair in two patients each.

**Conclusion:** Proximal humerus fractures treated with minimally invasive percutaneous plate osteosynthesis using locking compression plate with minimal soft tissue dissection, provides good functional outcome and early return of shoulder function.

## Introduction

Proximal humerus fracture is the second most common fracture of the upper extremity accounting for 45% of all humeral fractures^1,2^. The management of these fractures depends on the vascular status, bone quality, fracture pattern, degree of commination and patient factors. Non-operative management is preferred for elderly patients and those with major comorbidities and for undisplaced fractures^3^. However, treating these fractures using non-operative method requires high level of patient compliance and it is associated with complications like stiff shoulder and Sudeck’s osteodystrophy.

Fixation of these fractures is indicated when the greater tuberosity fragment displacement is >5mm, the shaft fragment displacement is >20mm, or the head fragment angulation is >45 degrees^3^. Various methods of fixation, including Kirschner wires, screws, conventional plate, antegrade nail and locking compression plate, have been well documented in the literature. With the advance of the design of locking system, those proximal humerus fractures with poor bone quality can be stably fixed. Minimally invasive percutaneous plate osteosynthesis (MIPPO) requires minimal soft tissue retraction and periosteal stripping and enables better preservation of the blood supply and improved healing of the fractures^4,5^. The MIPPO technique is now the preferred approach to treat periarticular and even certain diaphyseal fractures of long bones.

Here we present our experience of fixation with locking compression plate in complex proximal humerus fracture using the MIPPO technique and evaluation of the functional outcome.

## Materials and Methods

**Fig. 1:**
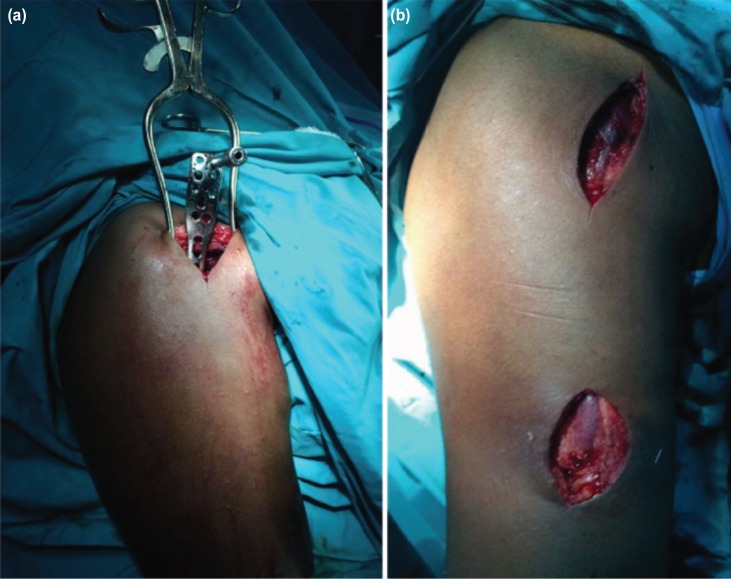
(a) Figure showing patient positioning and sliding in of the PHILOS plate. (b) Figure showing dual incisions after MIPPO technique.

Between 2013 and 2015, we treated 30 cases of proximal humerus fractures which presented to us by minimally invasive plate osteosynthesis using Proximal Humerus Internal Locking System (PHILOS). Patients above 18 years of age with proximal humerus fractures were included in this study. Although patients with associated lower limb fractures were included, patients with associated upper limb fractures, head injury and neurovascular injury were excluded. Fractures were classified according to Neer’s classification using plain radiographs. There were 23 patients with Neer’s Type 3 and seven patients with Neer’s Type 4 fracture pattern. All patients were operated within a week from the date of injury (range 1-6 days). Under general anaesthesia patients were positioned in beach chair on a radiolucent table with image intensifier used intraoperatively. Using dual incision technique, incision was made first along the deltopectoral groove proximally. Fibers of the deltoid and cephalic vein were retracted laterally and the conjoint tendon (short head of biceps and coracobrachialis muscle) medially. The fracture was reduced applying longitudinal traction along the arm with the shoulder in neutral rotation and reduction checked with the image intensifier. An appropriate size locking plate determined by the fracture geometry was slid in from proximal to distal position (Fig. 1a). The greater tuberosity and humeral head were stabilised provisionally with one or two Kirschner wires. If the lesser tuberosity was injured, it was pinned as well. To prevent impingement the tip of the plate was kept at the level of the proximal margin of the greater tuberosity. After confirming under image intensifier, we proceeded to fill locking screws proximally and a small incision was made for the placement of the distal screws (Fig. 1b). The wound was closed in layers. No drains were used with judicious use of local anaesthetic infiltration to suture margins for post-operative pain control.

Postoperative rehabilitation consisted of elbow flexion to 90 degrees and external rotation to 0 degrees for 3-4 weeks in order to reduce tension on the greater tuberosity and promote healing. Two days postoperatively, the patients were started on passive motion exercise with shoulder forward flexion to 45 degrees. One week postoperatively, the patients were encouraged to start passive mobilization of the shoulder with forward flexion to 60 degrees and external rotation to 10 degrees. Three to four weeks after the surgery, passive mobilization of the shoulder with forward flexion to 90 degrees and external rotation to 30 degrees was begun.

**Fig. 2:**
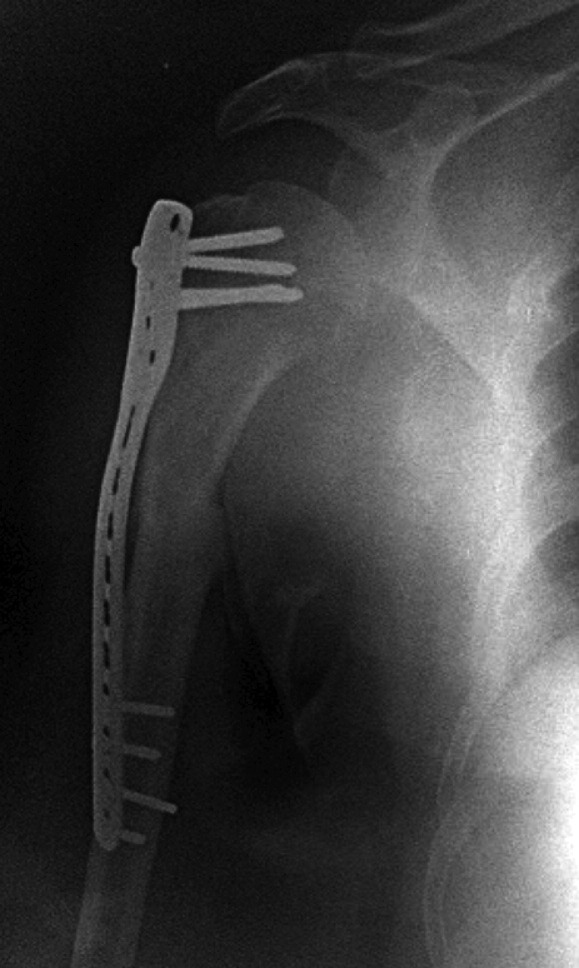
Post-op radiograph at 4 months showing good fracture union with implant *in situ*

**Fig. 3:**
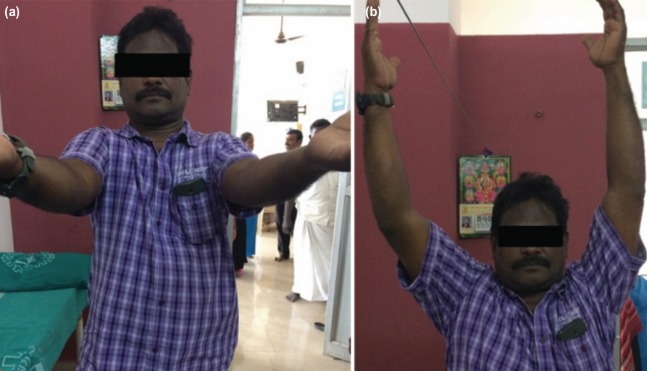
Follow-up at four months of right shoulder (a) Good forward flexion. (b) Good range of abduction.

Depending on the progress of healing as evident in the radiograph, patients were started on active exercise with forward flexion and external rotation at 5-6 weeks postoperatively. Seven to eight weeks postoperatively, patients commenced active shoulder joint internal rotation. Three months postoperatively, resistance exercise was encouraged. All patients were available for follow-up and were evaluated in the hospital at six weeks, three months, four months, six months and 12 months following surgery (Fig. 2). Patients were evaluated for the functional outcome, flexion and abduction range of motion (Fig. 3). Disabilities of the Arm, Shoulder and Hand (DASH) score was used for the functional outcome during review.

## Results

The study comprised of thirty patients with proximal humerus fractures with an average age of 58.8 years (range: 26 years to 65 years). All the fractures healed well with an average time of 13.2 weeks and all the patients were followed up to one year. The mean DASH score at one year follow-up was 8.69 (range: 2.50 to 17.16) (Table I). Twenty-six patients had excellent outcome, two good and two had fair outcomes at one year follow-up.

**Table I:** Functional outcome of all patients

SI. No	Age (years)	Type of fracture	Time to union (weeks)	DASH score	Flexion (degrees)	Abduction (degrees)
1	54	3 part	12	6.24	160	130
2	59	3 part	12	4.06	150	115
3	60	4 part	16	11.56	120	105
4	26	3 part	12	2.50	170	160
5	65	4 part	16	17.16	100	90
6	62	4 part	16	10.26	140	100
7	43	3 part	16	8.42	145	120
8	63	3 part	12	6.59	160	120
9	62	3 part	12	10.11	135	110
10	50	3 part	12	4.62	160	150
11	46	3 part	12	8.48	150	135
12	64	3 part	12	11.66	130	100
13	60	4 part	12	10.19	150	105
14	58	3 part	12	8.32	155	130
15	64	3 part	16	11.06	140	120
16	68	3 part	12	8.40	140	110
17	62	3 part	16	9.26	135	115
18	74	4 part	12	14.72	100	90
19	38	3 part	12	4.12	170	150
20	59	3 part	12	9.06	150	135
21	66	4 part	16	8.23	155	130
22	64	3 part	12	7.64	160	140
23	55	3 part	16	8.38	145	120
24	63	3 part	12	8.14	145	125
25	67	3 part	12	11.37	135	115
26	72	3 part	12	13.23	110	100
27	52	4 part	12	6.32	150	130
28	58	3 part	12	4.56	165	140
29	62	3 part	12	8.62	150	130
30	68	3 part	16	7.34	140	125

The average range of flexion of the shoulder at the follow-up was 143.8 degrees (range: 100 to 170 degrees) and the average range of abduction at the follow-up was 121.5 degrees (range: 90 to 160 degrees) (Table I). Three patients had superficial stitch abscess due to vicryl sutures and the sutures were removed after a short course of oral antibiotics. There were no complications of axillary nerve paraesthesia or deep infections in our study. We did not encounter any implant failure and avascular necrosis of humeral head at final follow-up (one year).

## Discussion

Proximal humerus fractures occur more commonly in elderly patients, with the mean age of 58.8 years in our study, comparable to the studies reported by Aarne *et al*^3^ and Lau *et al*^6^. These fractures commonly occur in osteoporotic bone and locking compression plate provides rigid fixation, increases torsional stiffness and fatigue resistance^7^, stability^8,9^, early mobilization and low failure rate^3^.

The delto-pectoral approach for proximal humerus fracture fixation is regarded as standard approach for adequate exposure^10^. Damage to the axillary nerve is very rare in this approach as compared to deltoid split approach where the axillary nerve injury is common with subsequent dysfunction of anterior deltoid^11,12^. The average DASH score in our patients with proximal humerus fracture treated with locking compression plate was 8.69, similar to the outcomes reported by Ismail *et al*^13^ and comparable to the report by Altmen *et al*^14^. The average range of flexion in our patient was 143.8 degrees (range: 100 to 170 degrees) and the average range of abduction of was 121.5 degrees (range: 90 to 160 degrees) which are similar to the outcomes reported by Ismail *et al*^13^ and Zu-Bin Zhou *et al*^15^.

The main advantages of MIPPO technique in fixation of complex comminuted fractures is preservation of fracture haematoma and surrounding soft tissue biology which would help in fracture healing. The proximal humeral locking plate can be used through this approach with good functional and radiological outcomes. Although the operating time is similar compared to conventional techniques, the advantages achieved in terms of preservation of soft tissue and fracture biology are the main advantages for this MIPPO technique.

The limitations in our study includes the small number of patients and short follow-up, limiting useful conclusions on late complications and long-term outcomes.

## Conclusion

Our study suggests that proximal humerus fractures treated with MIPPO using locking compression plate provides good functional outcome and viable option in enabling an early return of shoulder function. The delto-pectoral approach seems to be the best surgical route to treat using MIPPO technique with promising results.

## Conflict Of Interest

The authors declare no conflicts of interest.

## References

[1] Baron JA, Barrett JA, Karagas MR (1996). The epidemiology of peripheral fractures. Bone..

[2] Court-Brown CM, Garg A, McQueen MM (2001). The epidemiology of proximal humeral fractures.. *Acta Orthop Scand.*.

[3] Koljonen AR, Fang C, Lau TW, Leung F, Cheung NWK (2015). Minimally invasive plate osteosynthesis for proximal humeral fractures. J Orthop Surg (Hong Kong)..

[4] Rancan M, Dietrich M, Lamdark T, Can U, Platz A (2010). Minimal invasive long PHILOS®-plate osteosynthesis in metadiaphyseal fractures of the proximal humerus. Injury..

[5] Brunner A, Thormann S, Babst R (2012). Minimally invasive percutaneous plating of proximal humeral shaft fractures with the Proximal Humerus Internal Locking System (PHILOS). J Shoulder Elbow Surg..

[6] Lau TW, Leung F, Chan CF, Chow SP (2007). Minimally invasive plate osteosynthesis in the treatment of proximal humeral fracture.. *Int Orthop*.

[7] Weinstein DM, Bratton DR, Ciccone WJ, Elias JJ (2006). Locking plates improve torsional resistance in the stabilization of three-part proximal humeral fractures. J Shoulder Elbow Surg..

[8] Fakler JK, Hogan C, Heyde CE, John T (2008). Current concepts in the treatment of proximal humeral fractures. Orthopedics..

[9] Chudik SC, Weinhold P, Dahners LE (2003). Fixed-angle plate fixation in simulated fractures of the proximal humerus: a biomechanical study of a new device. J Shoulder Elbow Surg..

[10] Zlotolow DA, Catalano LW, Barron OA, Glickel SZ (2006). Surgical exposures of the humerus. J Am Acad Orthop Surg..

[11] Gardner MJ, Griffith MH, Dines JS, Briggs SM, Weiland AJ, Lorich DG (2005). The extended anterolateral acromial approach allows minimally invasive access to the proximal humerus.. *Clin Orthop Relat Res.*.

[12] Laflamme GY, Rouleau DM, Berry GK, Beaumont PH, Reindi R, Harvey EJ (2008). Percutaneous humeral plating of fractures of the proximal humerus: results of a prospective multicenter clinical trial. J Orthop Trauma..

[13] Ismail HD, Boedijono DR, Hidayat H, Simbardjo DS (2012). Minimal invasive plate osteosynthesis (MIPO) technique using anterolateral approach for treating closed proximal humerus fracture. Malays Orthop J..

[14] Altman GT, Gallo RA, Molinero KG, Muffly MT, Mascarenhas L (2011). Minimally invasive plate osteosynthesis for proximal humerus fractures: functional results of treatment. Am J Orthop (Belle Mead NJ)..

[15] Zhou ZB, Gao YS, Tang MJ, Sun YQ, Zhang CQ (2012). Minimally invasive percutaneous osteosynthesis for proximal humeral shaft fractures with the PHILOS through the deltopectoral approach. Int Orthop..

